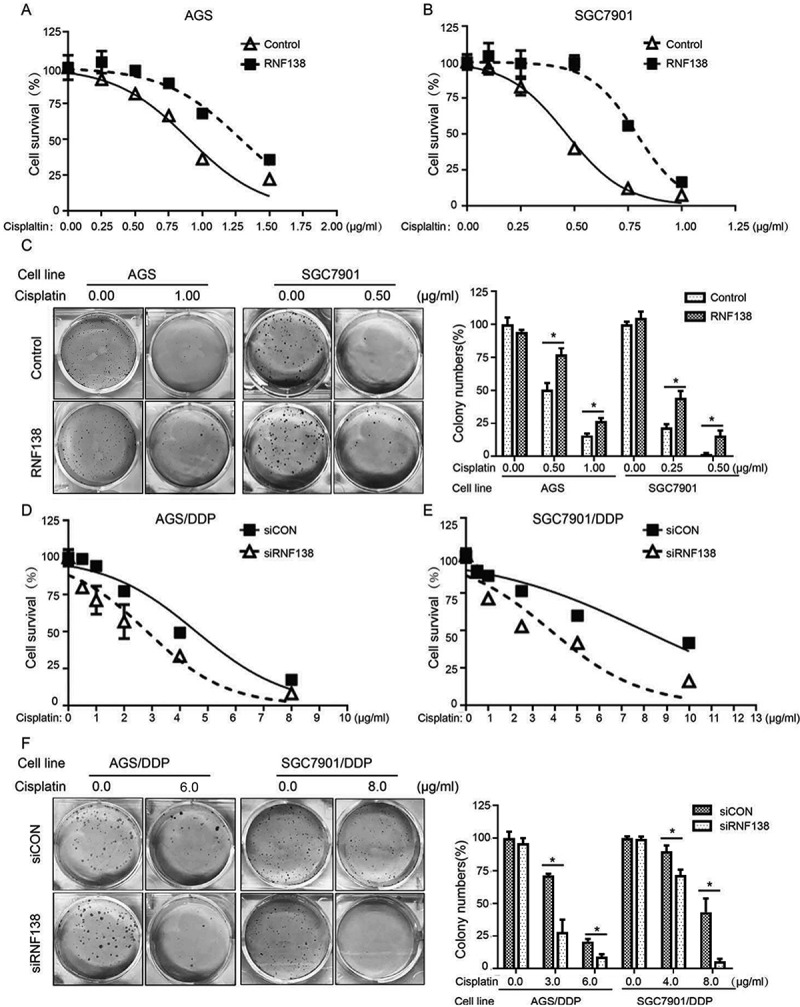# Correction

**DOI:** 10.1080/15384047.2024.2375109

**Published:** 2024-07-01

**Article title: RNF138 confers cisplatin resistance in gastric cancer cells via activating Chk1 signaling pathway**

**Authors**: Yalan Lu, Deqiang Han, Wenjie Liu, Rong Huang, Jinhuan Ou, Xiaoqiao Chen, Xizhe Zhang, Xuezhi Wang, Shijun Li, Lin Wang, Changzheng Liu, Shiying Miao, Linfang Wang, Changwu Ma and Wei Song

**Journal**: Cancer Biology & Therapy

**DOI**: https://doi.org/10.1080/15384047.2018.1480293

The authors recently noticed that in this article, the images in Figure 2C (AGS cells, left) and F (AGS/DDP cells, left) were inadvertently misplaced during the preparation of these figure. Fig. 2 is not supposed to be that one presented in the article. The amended version of this figure is now shown below.

**Correct Figure 2:**

**Figure:**